# *Schisandra chinensis* Bee Pollen Ameliorates Colitis in Mice by Modulating Gut Microbiota and Regulating Treg/Th17 Balance

**DOI:** 10.3390/foods13040585

**Published:** 2024-02-15

**Authors:** Ni Cheng, Xiaochao Wang, Yaoyao Zhou, Xuanxuan Zhao, Minghao Chen, Haoan Zhao, Wei Cao

**Affiliations:** 1 College of Food Science and Technology, Northwest University, Xi’an 710069, China; chengni@nwu.edu.cn (N.C.); 17835061568@163.com (X.W.); zhaohaoan@nwu.edu.cn (H.Z.); 2 Bee Product Research Center of Shaanxi Province, Xi’an 710065, China

**Keywords:** *Schisandra chinensis* bee pollen, colitis, FMT, gut microbiota, Treg/Th17 balance

## Abstract

Colitis is a chronic disease associated with alterations in the composition of gut microbiota. *Schisandra chinensis* bee pollen extract (SCPE) has been proved to be rich in phenolic compounds and effective in modulating gut microbiota, but its effect on colitis and the underlying mechanism remains unclear. This study investigates the relationship between colitis amelioration and the gut microbiota regulation of SCPE via fecal microbial transplantation (FMT). The results showed that administration of 20.4 g/kg BW of SCPE could primely ameliorate colitis induced by dextran sulfate sodium (DSS) in mice, showing as more integration of colon tissue structure and the colonic epithelial barrier, as well as lower oxidative stress and inflammation levels compared with colitis mice. Moreover, SCPE supplement restored the balance of T regulatory (Treg) cells and T helper 17 (Th17) cells. Gut microbiota analysis showed SCPE treatment could reshape the gut microbiota balance and improve the abundance of gut microbiota, especially the beneficial bacteria (*Akkermansia* and *Lactobacillus*) related to the production of short-chain fatty acids and the regulation of immunity. Most importantly, the protection of 20.4 g/kg BW of SCPE on colitis can be perfectly transmitted by fecal microbiota. Therefore, the gut microbiota–SCFAS–Treg/Th17 axis can be the main mechanism for SCPE to ameliorate colitis. This study suggests that SCPE can be a new promising functional food for prevention and treatment of colitis by reshaping gut microbiota and regulating gut immunity.

## 1. Introduction

Inflammatory bowel disease (IBD), mainly represented by ulcerative colitis and Crohn’s disease, affects more than 3.5 million people and has become a worldwide disease with a sharply rising incidence in this century [1]. Although the exact etiology of IBD remains unknown, the immune system, the disturbance of gut microbiota, and genetic susceptibility appear to be closely relevant in the pathogenesis of IBD [2]. Numerous research studies revealed that genetic factors alter gut homeostasis, triggering immune-mediated inflammation in genetically susceptible IBD individuals [3]. Gut microbiota determines gut homeostasis, gut barrier function, and immune responses in IBD development by microbial metabolites and their decomposition effect on the diet [4].

The increase in facultative anaerobes, including *Escherichia coli,* and the reduction of obligately anaerobes, the producers of SCFAs, were recognized as the common characteristics of gut microbiota in IBD individuals [5]. Notably, SCFAs such as acetate, propionate, and butyrate have been associated with IBD. These SCFAs could regulate the differentiation of T cells into Treg and Th17 and activate the intestinal mucosal immune system [6]. Growing research suggests that IBD is closely related to the immune system [7], and disruption of the Treg/Th17 balance can cause the occurrence of IBD [8]. Th17 cells specifically produce IL-17 and play a positive role in protection from IBD, but when immune regulation is dysfunctional, Th17 cells abnormally proliferate and produce a large number of pro-inflammatory cytokines, resulting in abnormal inflammatory pathology during infection and autoimmunity [9,10]. Although the accurate function of Treg cells is unknown, it is widely believed that Treg cells can reduce the risk of colitis by producing the inflammatory inhibitory effects of IL-10 [11]. Taken together, gut microbiota dysbiosis and the reduction of beneficial SCFAs disrupt the balance of Treg/Th17, which is an important cause of colitis.

Nowadays, despite the dedicated efforts of numerous experts in developing therapies for treating IBD, effective treatments for this recurrent disease are still lacking. The currently popular anti-colitis drugs are not only associated with side effects but also with a high cost. Therefore, it is necessary to develop natural products for treating IBD, ones that possess significant advantages including safety, low cost, and abundant availability [12]. Natural polyphenols, a kind of bioactive substance, have been proven to possess anti-inflammatory, antioxidant, anti-hypertensive, and anti-tumor properties [13]. The concept of the “three Ps” for gut health, which includes probiotics, prebiotics, and polyphenols, has shown the “prebiotic-like” effects of polyphenols [14]. Most dietary polyphenol, mainly existing in the form of oligomer and polymer, are poorly absorbed in the small intestine, and reach the colon to regulate the structure and function of gut microbiota. Foods rich in polyphenols, such as green tea, grapeseed, cocoa, and wine, have been reported to induce a decrease in the *Firmicutes*/*Bacteroidetes* ratio while enhancing the growth of *bifidobacteria* and *lactobacilli*, thereby contributing to a healthier gut microbiota composition [15]. On the other hand, polyphenols have been shown to ameliorate DSS-induced intestinal immune system disorders. For instance, rutin has been reported to reduce the proportion of CD4^+^T and CD8^+^T lymphocytes in colitis mice, exerting a protective effect against DSS-induced colitis [16]. Additionally, resveratrol can regulate the Treg/Th17 balance in mice with colitis in a dose-dependent manner [17,18].

*Schisandra chinensis* bee pollen collected by honey bees from the flowers of *S. chinensis* is rich in phenols and has been widely used as a healthy food source for centuries [19]. In our previous study, 12 phenolic compounds were identified in *S. chinensis* bee pollen extract (SCPE). The content of naringenin reached 1.89 mg/g and the total phenolic content was 101.83 mg GA/g. SCPE has shown the ability to modulate the gut microbiome of obese mice and prevent nonalcoholic fatty liver disease induced by a high-fat diet [20]. Pollen polyphenols seem to be a potential candidate for prebiotics. However, the effects of SCPE on the gut microbiota in colitis mice have not been researched, and the relationship between the amelioration of colitis and the modulation of gut microbiota by SCPE is not clear. Therefore, in this study, fecal microbiota transplantation (FMT) was used to investigate whether the improvement of colitis and intestinal immunity by SCPE was directly related to gut microbiota. Gut microbiota was removed through FMT from SCPE-fed mice to colitis mice. Finally, the effects of gut microbiota on host function were explored through the intestinal barrier, immune cells, inflammatory pathways, and SCFAs.

## 2. Materials and Methods

### 2.1. Preparation of SCPE

The sample of *S. chinensis* bee pollen, produced in Hubei province, was extracted three times by 75% (*v*/*v*) ethanol under heat reflux for 3 h. After centrifuging at 5000 rpm for 20 min, the supernatant was concentrated under vacuum to obtain *S. chinensis* pollen extract (SCPE).

### 2.2. Animal Experiment

**Figure 1:**
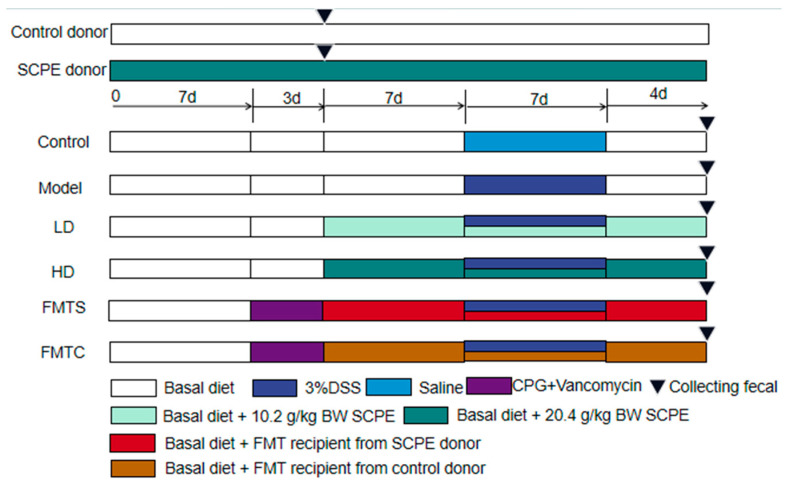
Experimental design.

Eighty male C57BL/6 mice (6 weeks old) were purchased from Xi’an Jiaotong University Laboratory Animal Center (laboratory animal production license number: SCXK 2018-001). These mice were kept in standard laboratory conditions and fed with standard rat food. Mice in the donor group were divided into two groups, namely Control donor group (n = 10) and SCPE donor group (n = 10), which were respectively gavaged with distilled water and 20.4 g SCPE/kg BW throughout the experiment. Other mice were divided into 6 groups (n = 10) and treated by the following procedures (Figure 1): The Control group and the model group were gavaged with distilled water. The LD and HD groups were gavaged with 10.2 and 20.4 g SCPE/kg BW respectively. The FMTS and FMTC groups were gavaged with fecal microbiota from the SCPE donor group and control donor group, respectively. The mice in the Model group, LD group, HD group, FMTS group, and FMTC group were given 3% (*w*/*v*) dextran sodium sulfate (DSS) in their drinking water for 7 days to induce chronic colitis. The mice in the control group were given the same concentration of saline. The status of mice in each group was observed and recorded, and the disease activity index (DAI) was calculated every day. The DAI was determined according to Chen et al. [21], including the score of stool consistency, weight loss, and blood in excreta. At the end of the experiment on the 18^th^ day, all mice were sacrificed, and the entire colon and spleen were harvested.

### 2.3. Fecal Microbiota Transplantation (FMT)

FMT was performed according to the protocols previously studied [22,23]. Mice in the FMTS and FMTC groups were given compound polyethylene glycol (200 mg/mL) in their drinking water and gavaged with vancomycin saline (200 mg/kg) for 3 days before the experiment to remove indigenous gut microorganisms. As shown in Figure 1, donor mice were fed with a basal diet or 20.4 g/kg BW of SCPE for 10 days, then their stools were collected daily in sterile conditions. Each stool sample (100 mg) was suspended in sterile saline (1 mL) and mixed well. The mixture was centrifuged and the supernatant was obtained for the following FMT. Mice in the FMTS and FMTC groups daily received 100 μL of fresh fecal microbiota supernatant from the SCPE and control donor groups, respectively, via gavage for 18 days.

### 2.4. Histological Analysis

Colon tissue was fixed with formaldehyde, then paraffin sections and stained with hematoxylin and eosin (H&E). Colonic structure was observed under the light microscope (BK-FL China, Zhengzhou, China) at a magnification of 200×. The degree of colon tissue damage was scored as follows. Cell inflammatory infiltration (0–3): from mild inflammatory cell infiltration in colon tissue to severe inflammatory cell infiltration; tissue damage (0–3): from colon tissue and crypts intact to serious colon tissue damage.

Immunohistochemical was performed as follow: the embedded colon sections described above were soaked in buffer (pH 6.0) and heated to antigen retrieval. Then colon tissue sections were treated with 3% H_2_O_2_ for 30 min. Staining was performed by immunohistochemical combination with antibodies against occludin and ZO-1 (1:100; Servicebio, Wuhan, China). Tissue sections were observed at a magnification of 400× and positive cells appear as brownish yellow cytoplasm. Optical density (OD) was measured by Image-Pro Plus 6.0 image analysis software using the images acquired from the immunohistochemistry experiments.

### 2.5. Measurement of Inflammatory Mediators

SOD, GSH-Px, MPO, MDA, and NO in the colon tissue were analyzed using commercial kits (Nanjing Jiancheng Bioengineering Institute, Nanjing, China). Serum levels of TNF-α, IL-1β, IL-18, IL-6, IL-17, and IL-10 were measured by commercially available ELISA kits (Fusheng Bio-Technology, Shanghai, China) following the producer’s manual protocols.

### 2.6. RNA Isolation and Quantitative Real-Time PCR

The total RNA of homogenized colonic tissues was separated using TaKaRa RNA separation kit (TaKaRa Biotech, Kusatsu, Japan). The cDNA templates were synthesized from equal amounts of total RNA using PrimeScipt™RT Master Mix (TaKaRa Biotech, Japan). The genes of mucin 2 (MUC2), toll-like receptors (TLR4), nuclear factor-kappa B (NF-κB), and inhibitor of NF-κB (IκB-α) were determined by qPCR. The forward and reverse sequences of qPCR primers were designed as below: β-actin: 5′-AGCTGCGTTTTACACCCTT-3′ and 5′-AAGCCATGCCAATGTTGTC-3′; MUC2: 5′-CTGACCAAGAGCGAACACAA-3′ and 5′-CATGACTGGAAGCAACTGGA-3′. TLR4: 5′-CAGTAGAAATGGCTTGAGTTTC-3′ and 5′-GGTTTCTGAGTGATAGGAATAC-3′; NF-κB: 5′-GTCTGCGTCAAGACTGCTAC-3′ and 5′-ACAAGTTCATGTGGATGAGG-3′; IκB: 5′-CGAGACTTTCGAGGAAATACC-3′ and 5′-GTCTGCGTCAAGACTGCTAC-3′. The cDNA templates were amplified with SYBR Green by quantitative real-time PCR instrument (Xi’an Tianlong Science and Technology Co., Xi’an, China). The values of gene expression were normalized with β-actin, and calculated by the formula 2^−ΔΔCt^.

### 2.7. Flow Cytometry Analysis

Blood was collected in the anticoagulant tube. Erythrocytes in the cell mass were fully lysed with erythrocyte lysate for 5 min, centrifuged at 1500 rpm for 10 min, and washed twice with PBS. Single cells were suspended in RPMI 1640 medium containing 10% fetal bovine serum to prepare single cell suspension. The spleen tissue of mice was ground in lymphocyte isolation fluid, and filtered into the petri dish with a disposable 70 μm filter. After centrifugation of spleen tissue homogenate, the white lymphocyte layer was aspirated, and erythrocytes were lysed with erythrocyte lysate. After washing with PBS, the single cells were resuspended in the medium to obtain the single lymphocyte suspension.

For CD4 and CD8 analysis, 10 μL of anti-CD3/CD4/CD8 antibodies (Biolegend, San Diego, CA, USA) was placed at the bottom of the flow tube and mixed with 100 μL cells under test, reacting in the dark at 4 °C for 30 min. After being washed with 2 mL of cold flow cytometry staining buffer, the cells were resuspended with 500 μL PBS for testing. Treg cells were analyzed by staining with anti-CD4 and anti-CD25 in the dark. The cells stained on the above surface were fixed and broken with Fix/Perm solution precooled for 30 min at 4 °C, then incubated with anti-Foxp3 antibody. Next, the cells were tested after resuspending with 500 μL PBS. For Th17 analysis, appropriate cell stimulants (PMA and ionmycin) and a protein transport blocker (Brefeldin A) were added to 200 μL cell suspension and incubated at 37 °C, 5% CO_2_ for 6 h. After washing the cells by PBS, they were stained with anti-CD4 antibody in the dark. Then, fixed with Fix solution and permeated with Perm solution in the dark, the cells were stained with anti-IL-17 antibody for 30 min. Cells were detected by flow cytometry (Agilent, Santa Clara, CA, USA) and events were recorded and analyzed with FlowJO software (Treestar, Ashland, OR, USA).

### 2.8. Microbiota Analysis

Fecal samples of mice in the donor groups and intestinal contents of mice in the experimental groups were collected and frozen in liquid nitrogen immediately. Total genomic DNA of gut microbiota was extracted from each samples using the Fast DNA isolation kit (AXYGEN, Shanghai, China). The quality of the DNA was detected by 0.8% agarose gel electrophoresis, and DNA was quantified by a Nanodrop spectrophotometer (Thermo Fisher Scientific, Waltham, MA, USA). The V3-V4 hypervariable regions of each sample 16S rRNA gene were amplified by PCR using V4R (CGGACTACHVGGGTWTCTAAT) and V3F (GGACTACHVGGGTWTCTAAT). The PCR sequence was 98 °C for 30 s, followed by 27 cycles at 98 °C for 15 s, 50 °C for 30 s, 72 °C for 30 s, and a final extension at 72 °C for 5 min. The integrity of the amplified PCR products was detected by 2.0% agarose gel electrophoresis. Target fragments were cut, then recovered using a gel recovery kit (Axygen, Union City, CA, USA). Using Quant-iT PicoGreen dsDNA Assay Kit for PCR, the product was produced in a Microplate reader (BioTek, FLx800, Winooski, VT, USA) and quantified, then mixed according to the amount of data required for each sample.

Qualified DNA fragments were analyzed by the Illumina MiSeq (Illumina, San Diego, CA, USA), then the quantitative insights into the microbial ecology (QIIME, v1.8.0) pipeline were employed to process the sequencing data to obtain a 200–450 bp V3–V4 16S sequence [24]. The operational taxonomy units (OTUs) were classified by QIIME software. Subsequently, rarefaction analysis and Shannon diversity index were calculated using UCLUST.

### 2.9. Measurement of Short-Chain Fatty Acid

The cecal contents of mice were collected and dissolved in distilled water equably. The supernatant was obtained by centrifuging at 4 °C, 13,000 r/min for 15 min. After mixing with 50% sulfuric acid at the ratio of 1:1, an appropriate amount of diethyl ether was added to the supernatant. Then, the sample was centrifuged again at 5000 r/min for 5 min at 4 °C. Lastly, the supernatant was extracted to measure the concentrations of short chain fatty acid by Gas Chromatography-Mass Spectrometer (8890/7000D, Agilent Technologies Co., Ltd.).

### 2.10. Statistical Analysis

All the data were determined in triplicate and the results were shown as the mean ± standard deviation. One-way analysis of variance (ANOVA) was performed using SPSS 20.0 software at *p* < 0.05. The data were graphically presented using GraphPad Prism 8.0.2 software. Different letters indicate significant differences between any two groups.

## 3. Results

### 3.1. SCPE Altered Composition of Gut Microbiota

**Figure 2:**
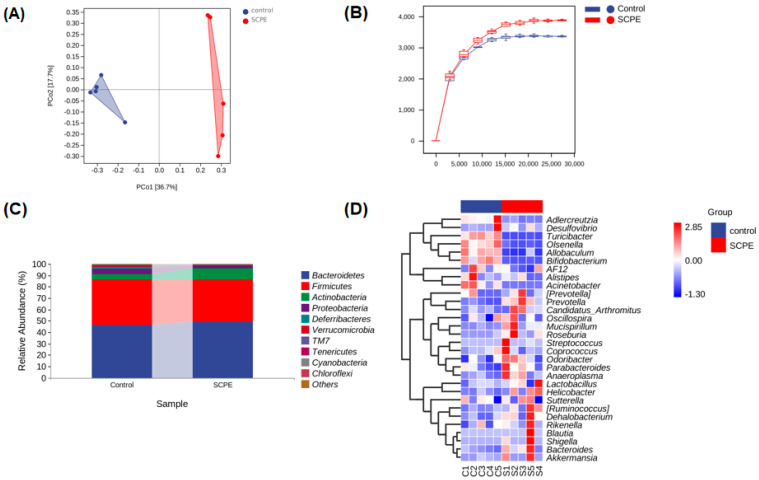
Effect of SCPE on gut microbiota composition. (**A**) PCoA of gut microbiota. (**B**) Sparse curve of gut microbiota. (**C**) Dominant bacterial relative abundance at phylum levels. (**D**) Heatmap analysis at genus level of gut microbiota.

In order to estimate the effect of SCPE on gut microbiota of donor mice, 16S rRNA pyrosequencing was carried out. Principal coordinate analysis (PCoA) reflects the similarity of sample composition. When the composition of microbiota of two samples is more similar, the projection distance of two points on the coordinate axis is closer. As shown in Figure 2A, compared with the control group, SCPE significantly treated altered gut microbial composition. The sparse curve predicts the total number and the relative abundance of each species at a given set of sequencing depths [25]. From Figure 2B, it demonstrated that the sample size of feces (n = 5) in this experiment was sufficient to reflect the diversity of microbiota in different groups of mice, and the gut microbial composition of SCPE-treated mice was more abundant than that of the control group.

Histograms (Figure 2C) showed that *Bacteroidetes*, *Firmicutes*, *Proteobacteria*, and *Actinobacteria* were the dominant phyla bacteria, accounting for about 94% of the total. Compared with the control group, the relative abundances of *Proteobacteria* and *Firmicutes* were decreased after SCPE treatment, but *Actinobacteria* and *Bacteroidetes* were increased significantly. As shown in Figure 2D, SCPE supplementation increased the abundance of *Roseburia*, *Akkermansia*, *Lactobacillus*, *Prepotella*, *Parabacteroides*, and *Mucispirillum* in the feces, while decreasing the abundance of *Allobaculum*, *Bacteroides*, and *Turicibacter*.

### 3.2. FMT from SCPE-Administration Mice Ameliorated DSS-Induced Colitis

**Figure 3:**
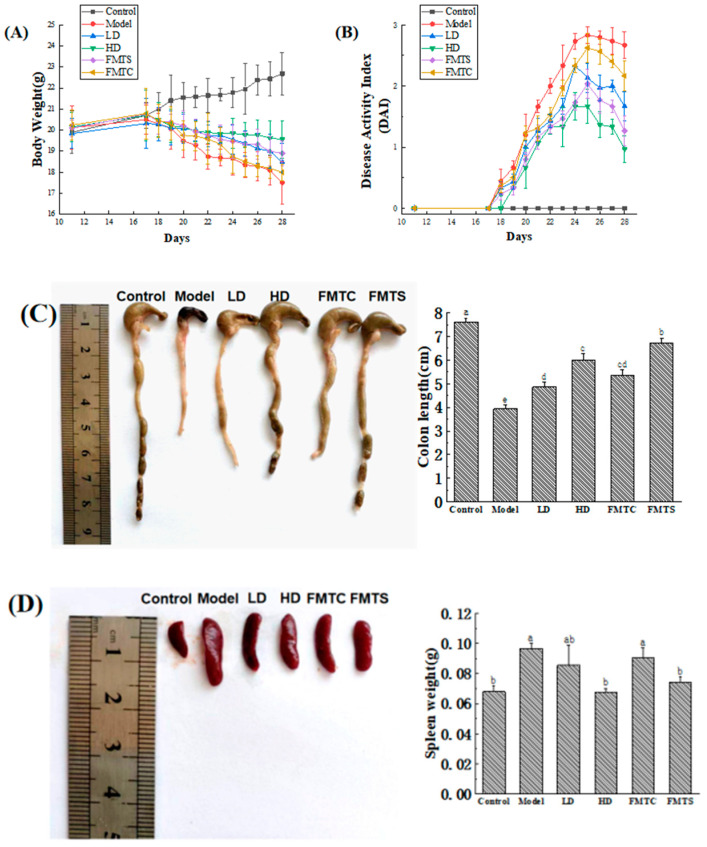
Effect of FMT from SCPE-administration mice on DSS-induced colitis. (**A**) Body weight. (**B**) Disease activity index (DAI). (**C**) Picture and length of colon. (**D**) Picture and weight of spleen. Different lower case letters indicate significant differences between any two groups.

Common symptoms of colitis include reduced food intake, diarrhea, and weight loss. As depicted in Figure 3A, the mice in the model group exhibited a significant decrease in weight after DSS consumption. Surprisingly, SCPE efficiently attenuated body weight loss. Mice in the FMTS group showed similar changes to those fed SCPE. Furthermore, on the third day of DSS consumption, mice in the model group displayed symptoms such as diarrhea, slow movement, and depression. In contrast, mice in the HD and FMTS groups did not exhibit these symptoms until the fourth or fifth day. DAI serves as a comprehensive indicator of disease severity, encompassing weight loss, diarrhea, and blood in the stool [26]. As shown in Figure 3B, the DAI index of mice in the model group increased immediately after DSS treatment. By the end of the experiment, the DAI index for the model group reached 2.67, which was 2.7 times higher than that of mice in HD group (0.97) and 2.1 times higher than that of mice in FMTS group (1.27).

Inflammation and congestion of the colon often lead to a shortened colon in mice with colitis. As an important immune organ, the spleen may become swollen due to immune disorder induced by DSS. Figure 3C,D demonstrate that administration of FMT (FMTC and FMTS groups) and SCPE (LD and HD groups) effectively alleviated colon shortening and spleen swelling, rendering them more similar to those observed in the control group.

### 3.3. FMT from SCPE-Administration Mice Improved the Gut Barrier

**Figure 4:**
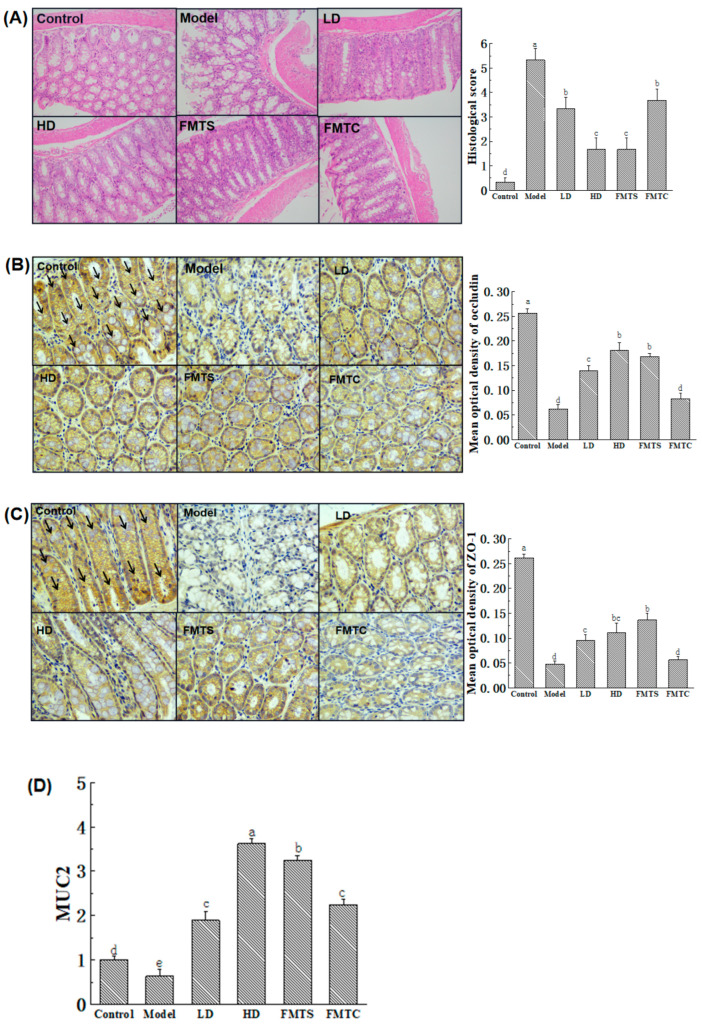
Effect of FMT from SCPE-administration mice on the gut barrier. (**A**) H&E-stained colon sections and semi-quantitative histological score. (**B**) ZO-1 and (**C**) occludin immunohistochemical section and score. (**D**) Expression of MUC2 gene in colon tissue. “→“ indicate positive antigen. Different lower case letters indicate significant differences between any two groups.

Histological analysis (Figure 4A) showed that the intestinal mucosa of the mice in the DSS-induced model group was severely damaged with scattered erosions and significant inflammatory infiltration, and a significantly higher histological score of 5.37 was observed. Conversely, less erosion was observed in most areas of the HD group, and the glandular structures were restored to some extent. The colonic tissues of the mice in the FMTS group were closer to those of healthy mice, with obvious crypt regeneration, intact muscle layer, and improved villi structure, resulting in a significantly lower histopathology score compared to the model group. Meanwhile, the FMTC group still had a small amount of erosion, an abnormal epithelial barrier, and a significantly higher histopathological score than that of the HD and FMTS groups.

Except for the histological analysis of the gut, the formation of tight junction-related proteins and multi-layer mucus barrier are also used to estimate the gut barrier. When tight junction-related proteins are damaged, gut permeability is increased [27]. As shown in Figure 4B,C, immunohistochemical analysis for intercellular tight junction-related proteins indicated that the expression of ZO-1 and occludin in the colon were strongly inhibited by 75.8% and 82.1%, respectively (*p* < 0.05), after DSS treatment. Excitingly, administration of different doses of SCPE (LD, HD groups) and FMTS increased the expression of ZO-1 by 124%, 192%, and 171%, respectively, compared to the model group (*p* < 0.05). Similarly, the expression of occludin was increased to 102%, 136%, and 191% in LD, HD, and FMTS groups compared with the model group (*p* < 0.05). Furthermore, due to the role of MUC2 as a major protein produced by the intestinal epithelium to protect the colon [28], we conducted q-PCR to detect the gene expression of MUC2. The result (Figure 4D) showed that DSS induced a 36% decrease in MUC2 expression compared with the control group. However, 10.2 and 20.4 g SCPE/kg consumption increased MUC2 expression 1.95 and 4.66 times compared with the model group. More importantly, FMT from SCPE mice and control mice showed a very good effect of promoting MUC2 gene expression.

### 3.4. Effects of FMT from SCPE-Administration Mice on Inflammation and Oxidative Stress

**Figure 5:**
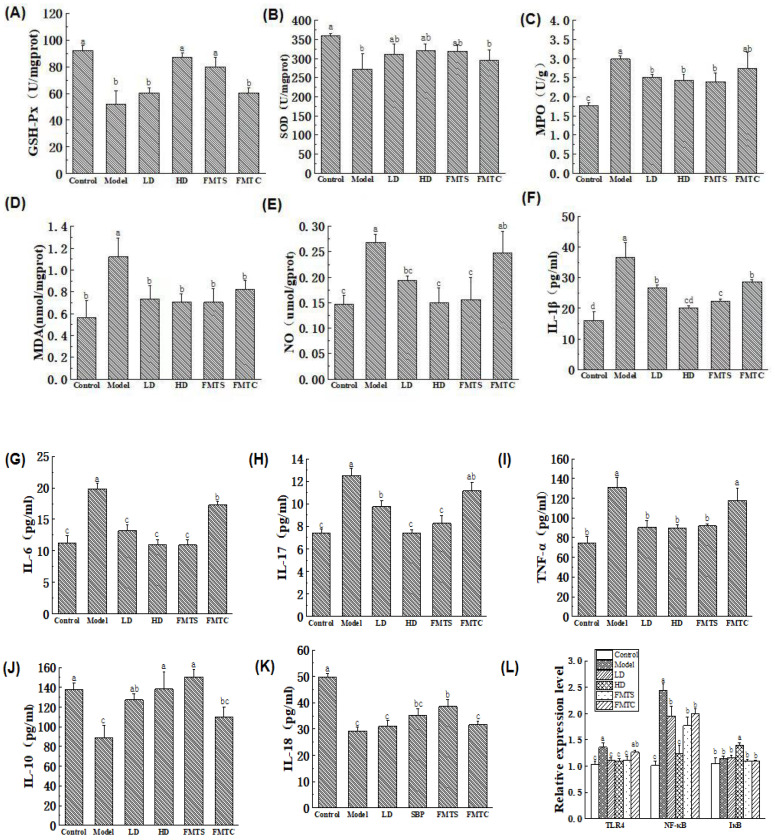
Effects of different treatments on oxidative stress. (**A**–**E**) GSH-Px, SOD, MPO, MDA, and NO. (**F**–**K**) Effect of different treatments on cytokine concentration IL-1β, IL-6, IL-17, TNF-α, IL-10, and IL-18. (**L**) Gene expression of TLR4, NF-κB, and IκB in colon tissue. Different lower case letters indicate significant differences between any two groups.

Oxidative stress usually interacts with inflammatory reactions. Therefore, we monitored oxidative stress and inflammatory cytokines during the progression of colitis. The activities of GSH-Px, SOD, and MPO, as well as the levels of MDA and NO in colonic tissue, were measured. As shown in Figure 5A–E, DSS administration significantly lowered the activities of GSH-Px and SOD, and improved the activity of MPO and the levels of MDA and NO (*p* < 0.05). Importantly, administration of 20.4 g/kg SCPE and FMTS significantly enhanced SOD activity, weakened the activity of MPO, and decreased the levels of MDA and NO (*p* < 0.05). In this study, the levels of IL-1β, IL-6, TNF-α, IL-10, IL-17, and IL-18 in serum were also examined, and the results were shown in Figure 5F–K. Treatment with SCPE and FMTS significantly suppressed the increase in pro-inflammatory cytokine levels (IL-1β, IL-6, and IL-17) and the decrease in anti-inflammatory cytokine levels (IL-10 and IL-18) induced by DSS.

To evaluate the effect of SCPE on the inflammatory pathway in DSS-induced colitis mice, we measured the mRNA expression of TLR, NF-κB, and IκB by q-PCR. The results (Figure 5L) showed that mRNA expression of TLR4 and NF-κB in colon tissue was significantly increased after treatment with DSS (*p* < 0.05). Compared with the model group, administration of different doses of SCPE (LD and HD groups) and FMTS ameliorated the expression of TLR4 by 18.09%, 19.46%, and 18.80% and the expression of NF-κB by 20.49%, 49.47%, and 27.57%, respectively. DSS did not influence the expression of IκB; however, gavage with 20.4 g/kg of SCPE (HD group) evidently promoted the expression of IκB (*p* < 0.05).

### 3.5. FMT from SCPE-Administration Mice Modulated the Response of Immune Cells

**Figure 6:**
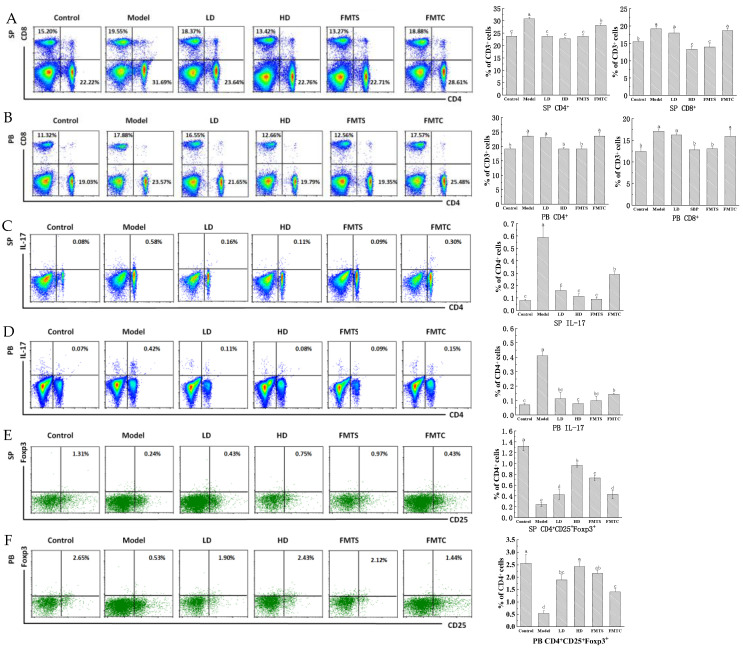
Effects of different treatments on the response of immune cells in peripheral blood (PB) and spleen (SP). CD4^+^ T and CD8^+^T in (**A**) SP and (**B**) PB, as well as (**C**–**F**) Th17 (CD4^+^IL17^+^) and Treg (CD25^+^Foxp3^+^) cells in SP and PB. Different lower case letters indicate significant differences between any two groups.

The abnormal activation of immune cells, which promotes the production of inflammatory cytokines, has been reported to trigger colitis [29]. The results of flow cytometry analysis showed that compared with the model group, 20.4 g/kg of SCPE treatment (HD group) and FMTS significantly downregulated the proportion of CD4^+^T and CD8^+^T lymphocytes in the peripheral blood (PB) and spleen (SP) of colitis mice (*p* < 0.05), although DSS administration induced marked upregulation of them. More importantly, there was no significant difference between the HD group and the FMTS group (Figure 6A,B).

Th17 and Treg cells are subsets of CD4^+^T cells. Th17 cells are believed to play a pathogenic role, while Treg cells have an obvious effect on anti-inflammation and immunomodulation [30]. To elucidate effects of SCPE and FMT treatment on CD4^+^ T cell differentiation, Th17 and Treg cells were analyzed by flow cytometry. The results showed (Figure 6C–F) that compared with the control group, the model group exhibited a significant increase in the proportion of Th17 cells and a significant decrease in the proportion of Treg cells (*p* < 0.05). Surprisingly, the percentages of Th17 and Treg cells in peripheral blood and spleen of mice treated with SCPE and FMT were significantly restored (*p* < 0.05). In comparison to the model group, the HD group exhibited a decrease of 80.95% (PB) and 81.03% (SP) in the proportion of Th17 cells, while the FMTS group showed a decrease of 81.03% (PB) and 84.48% (SP). In the HD group, the proportion of Treg cells increased by 78.19% (PB) and 75.00% (SP), while in the FMTS group, it increased by 75.26% (PB) and 68.00% (SP) compared with the model group. These results showed that, after treatment with FMTS, the proportion of Treg cells was increased and the proportion of Th17 cells was decreased, which was better than the treatment of FMTC.

### 3.6. FMT from SCPE-Administration Mice Improved Gut Microbial Structure and Function

**Figure 7:**
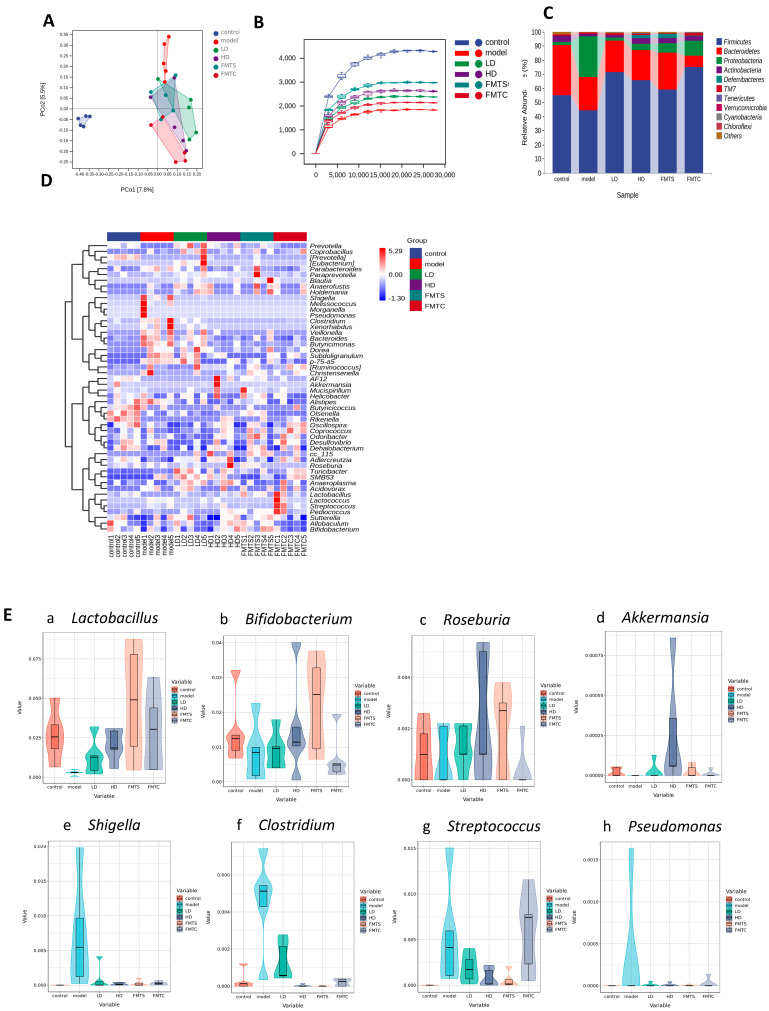
Effects of different treatments on gut microbial structure and function. (**A**) Principal coordinate analysis. (**B**) Sparse curve. (**C**) Relative abundance (%) of bacterial at phylum level in six groups. (**D**) Heatmap analysis at genus level. (**E**) Differences in relative abundance of gut microbiota at genus level.

The composition of gut microbiota in mice after FMT treatment was studied to further understand the effect of gut microbiota on colitis. From Figure 7A, PCoA revealed that the HD and FMTS groups have similar composition of gut microbiota. As shown in Figure 7B, DSS reduced the richness of gut microbiota, while treatment of FMTS and SCPE recovered gut microbiota. Compared with the model group, the abundance of *Firmicutes* in SCPE-administration mice (LD and HD groups) was increased, and the abundance of *Proteobacteria* was decreased, which was similar to that of FMTS (Figure 7C). As shown in Figure 7D, the heatmap at the genus level of gut microbiota, *Pseudomonas*, *Bacteroides*, *Clostridium*, *Duroa*, *Streptococcus*, *Mucispirillum*, and *Shigella* were significantly increased in the model group, whereas *Lactobacillus*, *Akkermansia*, *Oscillospira*, *Bifidobacterium*, *Prevotella*, *Roseburia, Coprococcus*, *Odoribacter*, *Coprobacter*, *Blautia*, and *Eubacterium* were significantly reduced. Surprisingly, the gut microbiota recovered after treatment with SCPE and FMTS.

At the genus level, the relative abundance of gut microbiota was further analyzed among the six groups. As shown in Figure 7E, DSS inhibited the relative abundance of *Lactobacillus* (a), *Bifidobacterium* (b), *Roseburia* (c), and *Akkermansia* (d) in the model group. SCPE at 20.4 g/kg BW and FMTS obviously alleviated the inhibition of those bacterial genera. The relative abundance of *Lactobacillus* in HD group increased to six times higher than that of model group, and FMTS group increased to sixteen times higher than that of model group. Additionally, *Shigella* (e), *Clostridium* (f), *Streptococcus* (g), and *Pseudomonas* (h) were increased significantly in the model group, but reduced in the SCPE-treated group and FMTS group. The relative abundance of *Shigella* in the HD group was reduced to 1/43 of that in the model group, and FMTS group was reduced to 1/27 of that in the model group.

### 3.7. FMT from SCPE-Administration Mice Promoted the Concentration of SCFAs in the Cecum

To further clarify the relationship between the metabolites of gut microbiota and colitis, we determined the concentration of acetic acid (AA), propionic acid (PA), butyric acid (BA), and valerate acid (VA) in the cecum of mice. As shown in Table 1, DSS reduced the SCFAs concentration. Among these, PA was reduced by 58.73% and BA by 72.59% compared to the control group. Conversely, SCPE and FMT treatment restored SCFAs in the cecum of mice. Especially compared with the model groups, treatment with 10.2 g/kg and 20.4 g/kg of SCPE (LD and HD groups), as well as FMTS, significantly increased the BA contents in the cecum by 1814%, 249%, and 346%, respectively (*p* < 0.05). The results indicated that SCPE may enhance the concentration of SCFAs by regulating the construction of the gut microbiota.

## 4. Discussion

In our previous study, SCPE was analyzed to contain 12 phenolic compounds, in which naringenin, chrysin, rutin, and isoliquiritigenin were the main compounds [20]. Naringenin is a naturally occurring phenolic compound with anti-inflammatory activity [31] and promotes recovery from colonic damage through suppression of epithelial TNF-α production [32]. Chrysin is also verified to own the inflammatory activity [33] and can ameliorate colitis by modulating the PXR/NF-kappaB signaling pathway [34]. Furthermore, rutin can be storage in intestinal mucosa to extend anti-inflammatory activity in colitis [35]. Therefore, we suppose that SCPE can attenuate colitis at least by anti-inflammatory activity in DSS-induced colitis mice. More importantly, we have demonstrated that bee pollen polyphenols alleviate DSS-induced colitis and regulate the composition of gut microbiota in colitis mice [21]. However, it has not been verified whether this gut protection can be attributed to the microbiota. In this study, we mainly explored the effect of gut microbiota on SCPE remitting colitis. The results of this study demonstrate that supplementation with SCPE was advantageous in increasing the diversity of gut microbiota in mice, as well as enhancing the relative abundance of *Roseburia*, *Akkermansia*, *Lactobacillus*, *Prepotella*, *Parabacteroides*, and *Mucispirillum*. Dietary polyphenols can selectively increase the proportion of beneficial bacteria, optimize the structure of gut microbiota, and have positive implications for health [36]. Based on this, we further performed FMT to explore the role of gut microbiota in colitis treated with SCPE.

We observed that administration of both 20.4 g/kg of SCPE and FMT from 20.4 g/kg of SCPE-administration mice (FMTS) significantly alleviated colitis, showing as lower DAI, longer colon, better gut barrier, lower inflammation and oxidative stress, and more balanced immunity system and gut microbiota compared to the model group. Therefore, we conclude that SCPE ameliorates DSS-induced colitis via modulating gut microbiota. In present studies, FMTS could increase the abundance of *Lactobacillus, Bifidobacterrium, Roseburia*, and *Akkermansia*, while decreasing the abundance of *Shigella*, *Clostridium*, *Streptococcus*, and *Pseudomonas* of colitis mice.

SCFAs are the key production of gut microbiota for promoting human health. Changes in the SCFAs levels in the gut may be diagnostic biomarkers of IBD. *Bifidobacterium*, *Lactobacillus*, and *Clostridium* can ferment dietary fiber to produce SCFAs, especially butyrate [37], which is closely related to the development of colitis [38]. As reported, the decrease in the human *Roseburia* was observed in patients with CD and UC [39,40], because *Roseburia* is a converter of AA to BA. The reported researches reveal that BA attenuates intestinal inflammation and improves intestinal barrier function in infected mice [41]. SCFAs, as an important energy source, play an important role in regulating the intestinal epithelial cells growth and differentiation, as well as maintaining the barrier and defense function of the gut epithelium [42]. When the gut barrier is broken, the bacteria come into direct contact with intestinal epithelial cells, leading to the breakdown of the balance between gut microbiota and the immune system, and further resulting in colitis [43]. In our study, SCPE and FMTS administration increased cecal BA contents and facilitated the recovery of gut epithelial structure in colitis mice. The structure of colonic tight junction proteins ZO-1 and occludin of mice recovered, and the expression of MUC2 mRNA increased significantly after being treated with FMTS. The results were in agreement with the research of Rescigno et al. that the gut microbiota and its metabolite SCFAs can stimulate an increase in MUC2 mRNA in the colon [44]. Therefore, SCPE and FMTS showed superior regulation of gut microbiota and produced more SCFAs for the recovering gut structure of colitis mice.

**Figure 8:**
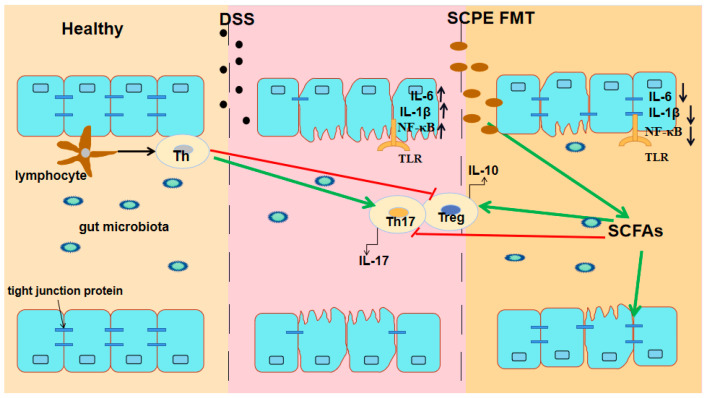
SCPE treated colitis mice via the mechanism of “gut microbiota- SCFAS- Treg/Th17”.

The immune response is considered to be a key factor in the development of colitis. The variety of immune cells regulate immune response and simultaneously activate downstream signaling pathways to produce cytokines, which promote the progression of colitis [9]. As reported, the proportion of circulating CD4^+^T and CD8^+^T lymphocytes increased in patients with IBD [45]. Th17 and Treg cells, subsets of CD4^+^T cells, have been recently identified as immune cells closely associated with colitis [46]. Th17 cells release IL-17, which is a cytokine causing colitis by increasing production of chemokine to recruit neutrophils and monocytes to the site of inflammation [47]. Although the exact function of Treg cells is unknown, it is widely believed that Treg cells can reduce the risk of colitis by producing the anti-inflammatory cytokine IL-10. IL-10 is able to suppress T cell-mediated immune response and ameliorate colitis by inhibiting antigen presenting cells, and downregulate IL-1β, IL-6, and TNF-α secreted by macrophages and T cells [11]. In our study, the proportions of CD4^+^T and CD8^+^T lymphocytes in the peripheral blood and spleen were reduced after SCPE and FMTS administration, while the proportions of Th17 cells were decreased and of Treg cells were increased, significantly. Meanwhile, SCPE and FMTS administration significantly suppressed the expression of pro-inflammatory cytokines (IL-1β, IL-6, and IL-17) and TNF-α, and enhanced the expression of anti-inflammatory cytokines (IL-10 and IL-18). It was evident that SCPE and FMTS administration could regulate the expression of inflammatory cytokines and consequently alleviate DSS-induced colitis in mice. *Bifidobacterium* and *Lactobacillus* can promote the increase in the proportion of Treg cells in mice [48]. Similarly, another study confirmed that the *Bacteroides fragilis* improved colitis in mice by inhibiting IL-17 production [49]. *Akkermansia* is also a recently discovered strain that prevents IBD. It not only improves the gut barrier by interacting with Toll-like receptor 2, but also induces IgG homeostasis and antigen-specific T cell response in mice, which improves DSS-induced UC mice [50,51]. In addition, *Lactobacillus* can decompose tryptophan into indole-3-acetaldehyde and indole-3-acetic acid, which can activate the immune system and keep the integrity of gut barrier [52]. These results indicated that SCPE-caused variation of gut microbiota played a crucial role in the amelioration of DSS-induced colitis mice. More importantly, the main mechanism may be the regulation of immune cells (Treg/Th17) by the gut microbiota via its metabolites SCFAs (Figure 8).

## 5. Conclusions

In conclusion, this research illustrates the protective mechanism of SCPE in DSS-induced colitis. SCPE can modulate the composition of gut microbiota in DSS-induced colitis mice, increase the richness of gut microflora, enrich the beneficial bacteria (*Akkermansia* and *Lactobacillus),* increase the production of SCFAs, improve the mucosa barrier function, cut down the proportions of CD4^+^T and CD8^+^T lymphocytes, decrease the proportions of Th17 cells, increase the proportions of Treg cells, inhibit the expression of Th17 cytokines, and reduce IL-17 levels. More importantly, the protection of SCPE could be transmitted through fecal bacteria transplantation. Therefore, modulation of gut microbiota and regulation of Treg/Th17 balance may be the major mechanism of SCPE amelioration colitis.

## Figures and Tables

**Table 1:** Content of short-chain fatty acids in cecum.

Group	SCFAs Concentrations (mg/g)			
	AA	PA	BA	VA
Control	1.59 ± 0.12 ^a^	0.63 ± 0.09 ^a^	1.35 ± 0.53 ^ab^	0.12 ± 0.04
Model	0.62 ± 0.30 ^b^	0.26 ± 0.11 ^b^	0.37 ± 0.06 ^c^	0.08 ± 0.05
LD	1.05 ± 0.33 ^a^	0.27 ± 0.14 ^b^	1.04 ± 0.58 ^ab^	0.09 ± 0.01
HD	1.40 ± 0.31 ^a^	0.37 ± 0.11 ^ab^	1.29 ± 0.41 ^ab^	0.10 ± 0.03
FMTS	1.40 ± 0.63 ^a^	0.42 ± 0.15 ^ab^	1.65 ± 0.51 ^a^	0.12 ± 0.04
FMTC	1.00 ± 0.46 ^a^	0.29 ± 0.08 ^b^	0.49 ± 0.33 ^c^	0.08 ± 0.02

The data in the table were shown as the mean ± standard deviation. Different lower case letters indicated significant differences between any two groups.

## Data Availability

The original contributions presented in the study are included in the article, further inquiries can be directed to the corresponding author.
